# Survival and predictors of mortality after completion of TB treatment among people living with HIV: a 5-year analytical cohort

**DOI:** 10.1186/s12879-023-08217-9

**Published:** 2023-04-18

**Authors:** Ivan Lumu, Joseph Musaazi, Aggrey Semeere, Ian Handel, Barbara Castelnuovo

**Affiliations:** 1 Infectious Diseases Institute - College of Health Sciences, Makerere University, P.O. Box 22418, Kampala, Uganda; 2 Edinburgh Medical School, University of Edinburgh, Edinburgh, United Kingdom

**Keywords:** Survival, Tuberculosis, HIV, Antiretroviral therapy, TB treatment completion

## Abstract

**Background:**

After completion of TB treatment patients may remain at risk of co-morbidity and mortality. We determined the survival and predictors of all-cause mortality after completing TB treatment among ART-experienced patients.

**Methods:**

This was a retrospective cohort analysis of all ART experienced patients who completed TB treatment at a specialist HIV clinic in Uganda, between 2009 and 2014. The patients were followed for five years after TB treatment. We determined the cumulative probability of death, and predictors of mortality using Kaplan-Meier methods and Cox proportional hazard models, respectively.

**Results:**

A total 1,287 patients completed TB treatment between 2009 and 2014, of which 1,111 were included in the analysis. At TB treatment completion, the median age was 36 years (IQR: 31–42), 563 (50.7%) were males, and median CD4 cell count was 235 cells/mL (IQR: 139–366). The person-time at risk was 4410.60 person-years. The all-cause mortality rate was 15.42 (95% CI: 12.14–19.59) per 1000 person-years. The probability of death at five years was 6.9% (95%CI: 5.5- 8.8). In the multivariable analysis, CD4 count < 200 cells/mL was a predictor of all-cause mortality (aHR = 1.81, 95%CI:1.06–3.11, p = 0.03) alongside history of retreatment (aHR = 2.12, 95%CI: 1.16–3.85, p = 0.01).

**Conclusion:**

Survival post TB treatment in ART experienced PLHIV is reasonably good. Most deaths occur within two years after TB treatment completion. Patients with a low CD4 count and those with a history of retreatment have an increased risk of mortality which underscores the need for TB prophylaxis, detailed assessment, and close monitoring after completion of TB treatment.

**Supplementary Information:**

The online version contains supplementary material available at 10.1186/s12879-023-08217-9.

## Background

Tuberculosis(TB) is responsible for most AIDS-related deaths accounting for 214,000 deaths globally among People living with HIV (PLHIV) in 2020. [1] People living with HIV are about five times more likely to develop TB as compared to HIV negative individuals, and the risk increases as the disease advances. [2, 3] This risk, coupled with a high HIV prevalence in sub Saharan Africa (sSA) is driving the TB epidemic further, despite the availability of effective TB treatment. [3] Moreover, most TB deaths occur during the first three months of TB treatment initiation. [4] Mortality during this period has been associated with low CD4 counts, not being on antiretroviral therapy (ART) [5, 6] and low body mass index (BMI) [4] at the time of TB diagnosis. Some studies in HIV negative patients indicate that even after successful completion of TB treatment, up to 59% of patients get lung impairment, [7] and consequently chronic lung disease. [8] Moreover, some observational studies have reported reduced long term survival in TB survivors in the United State [9] and Vietnam. [10] The pooled standardised mortality after TB treatment in these studies is about three-fold higher than the general population. [11] Death in these cohorts has been attributed to malignancies, cardiovascular complications, bacterial pneumonia, and TB reinfection. [10–12] Similarly, reduced long term survival has been reported among PLHIV co-infected with TB at the time of entry in care in the UK, and Latin America. [13, 14] However, literature on long term survival after successful TB treatment in HIV and TB high burden settings is scarce. In the era of ART, such evidence on the long term prognosis and timing of these deaths would be important for HIV programs and clinics and would guide the designing of interventions to improve survival. Therefore, this study aimed to investigate the long-time survival of ART experienced patients who complete TB treatment and to determine the predictors of mortality after TB treatment.

## Methods

### Study design

We retrospectively studied a cohort of PLHIV who completed TB treatment between 1st January 2009 and 31st December 2014 at the Infectious Diseases Institute, Makerere University, Uganda.

### Setting

The Infectious Diseases Institute clinic has registered over 33,000 patients since its inception, and approximately 8000 patients are currently active in care. In 2009, the clinic adopted the WHO model of providing integrated care for HIV and TB in the same clinic to improve treatment outcomes. [15] Patients’ care at the clinic follows the national TB and HIV treatment guidelines which are closely aligned with the WHO treatment guidelines. During the study period, new cases with presumed Drug-Sensitive Pulmonary TB (DSPTB)received an intensive phase of Isoniazid, Rifampicin, Pyrazinamide and Ethambutol daily for two months and a continuation phase of either Isoniazid and rifampicin daily for four months or Isoniazid and Ethambutol daily for six months. However, the continuation phase might be longer depending on the anatomic site of the disease. Currently, all DSPTB cases are given only the four-months continuation phase. The quality control department regularly verifies the quality of patients’ records and updates the follow-up status of the patients. Patients’ follow-up status is maintained by active tracing and tracking of patients with missed appointments. Patients that have missed their appointment and cannot be traced after two weeks from the appointment date are considered lost to follow-up.

### Participant eligibility

This study included all adults PLHIV aged ≥ 16 years who had completed TB treatment between 1st January 2009 and 31st December 2014. We excluded patients who were not taking lifelong ART during this period since lifelong ART is a known mortality modifier in PLHIV. [16, 17]

### Definitions and procedures

Clinical diagnosis means a patient had radiological and/or clinical evidence of active TB and was treated for TB without bacteriological evidence. Microbiological diagnosis means the patient had bacteriological evidence of TB in the collected specimen. Extra pulmonary TB means the patient is diagnosed with TB involving other organs without evidence of the disease in the lungs. Pulmonary TB means that the patient is diagnosed with tuberculosis that involves the lungs or broncho-tracheal tree.

Completion of treatment refers to any patient with clinical or microbiology evidence of tuberculosis that was started on anti-TB drugs and discharged as either ‘cured’ or ‘completed’ after taking the full course of the prescribed treatment.

The date of TB treatment completion was used as the time of entry into the study. For patients that had more than one episode, we considered the entry point to be the date of treatment completion for the last episode provided they completed treatment during the study period. Patients were then followed-up from the date of TB treatment completion to the date of the last visit in the event of death, transfer, or loss to follow up. Patients that were alive after five years of follow-up were administratively censored.

### Study outcome

The primary outcome was the time to all-cause mortality after successful TB treatment. Secondary outcomes were; cumulative probability of death and mortality rate at one, three years and five years.

### Statistical analysis

Descriptive analysis was done using frequencies and percentages for categorical variables or median and interquartile ranges (IQR) for continuous variables. Mortality rates were estimated using Poisson methods. Probabilities of deaths were estimated using Kaplan-Meier methods and compared using the Log-rank test. We examined the association between sex, age, calendar year of TB diagnosis, type of TB diagnosis, TB history, anatomical site of TB, WHO HIV clinical stage, and CD4 cell count, Body Mass Index, ART duration, ART regimen type, and time to all-cause mortality using Cox proportional hazard (Cox PH) regression model. We reported both unadjusted and adjusted Hazard ratios (uHR and aHR). All baseline characteristics (Table 1) were examined if they were associated with mortality. The factors which had a *p*-value < 0.2 at unadjusted models were entered into the multivariable model (performed on complete cases) to adjust for confounding. However, each of the factors that had *p* value > 0.2 at unadjusted analysis, were returned into the multivariable model one by one and then dropped if they had an adjusted *p*-value > 0.2. Statistical significance was set at an alpha of 0.05. The *p*-value and 95% confidence interval are reported. We checked for; Cox proportional hazard assumption using Schoenfeld residuals, period effect by the inclusion of calendar year of TB diagnosis as a covariate in the Cox PH model, effect modification by fitting interactions between some covariates, multi-collinearity using variance inflation factor method, and accounted for heterogeneity using robust standard errors in Cox PH model to account for variation across different calendar years. In the sensitivity analysis, the effect of loss to follow-up was examined using the worst-case scenario assumption that all patients lost to follow-up were dead, at time of loss to follow-up, as has been reported in some cohorts. [18] Also, sensitivity analysis was performed considering all patients lost to follow-up be alive at five years and in care elsewhere (best-case scenario). The effect of missing data on covariates was examined by refitting the final multivariable model using multiply imputed values from multiple imputations using chained equations with 20 imputations, which were selected based on the proportion of missing values. All the statistical analysis was performed using STATA software (version 16.1).

## Results

### Participants’ characteristics

**Fig. 1:**
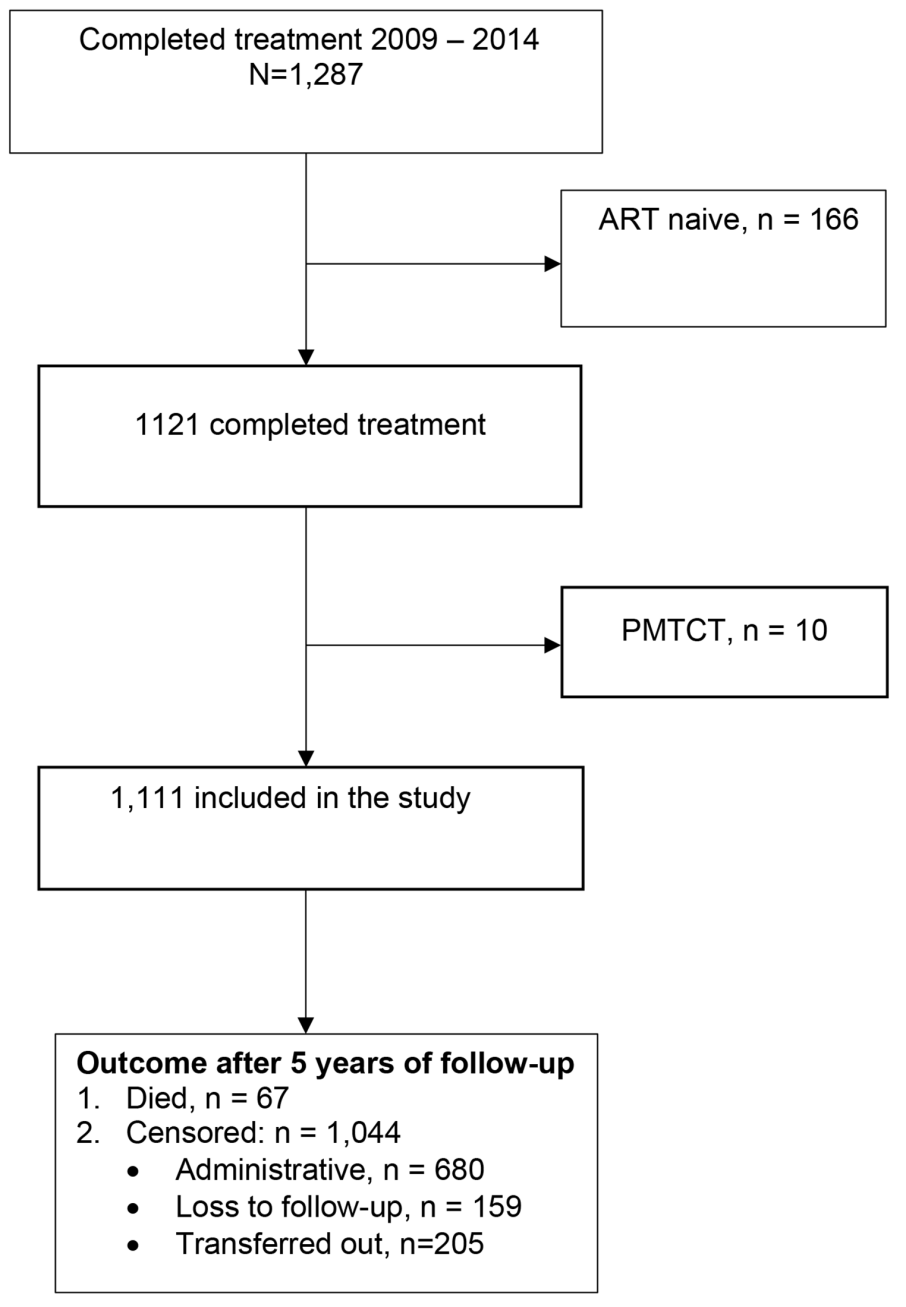
Patients’ inclusion process and outcomes during follow up

A total of 1,287 PLHIV patients completed TB treatment from 2009 to 2014. Of these 166 were ART naive and 10 patients were on ART temporarily as prevention of mother to child transmission (PMTCT), and these were excluded from the analysis. A total of 1,111 patients were included in the analysis and Fig. 1 shows the inclusion process and patients’ outcomes after follow-up.

For the 1,111 patients, the median age was 36 years (IQR: 31–42), 563 (51%) were males, median CD4 counts was 235 cells/mL (IQR: 139–366), and 393 (42%) had CD4 counts below 200 cells/ mL at TB treatment completion. Six hundred and forty-nine (59.2%) had been diagnosed with pulmonary TB, 155 (14.0%) had a history of previous TB treatment, and the majority (92.2%) were on first-line ART at TB treatment completion. The distributions of other patients’ characteristics are presented in Table 1.

**Table 1:** Participants’ characteristics at TB diagnosis or TB treatment completion

Characteristics	Number (%) N = 1,111
*Characteristics at TB treatment completion*	
Sex	
Female	548 (49.3%)
Male	563 (50.7%)
Age in years, median (IQR)	36 (IQR 31, 42)
16_24	74 (6.7%)
25_34	396 (35.6%)
35_44	449 (40.4%)
45+	192 (17.3%)
CD4 count (cells/mL) median (IQR )	235 (IQR:139, 366)
<200	393 (42.4%)
>=≥200	533 (57.6%)
Body mass index (Kg/M^2^), median (IQR)	22.0 (IQR 20.1, 24.2)
< 18	86 (8.2%)
≥ 18	964 (91.8%)
HIV WHO clinical stage	
Stage III	463 (41.9%)
Stage IV	642 (58.1%)
ART type	
1st line	1024 (92.2%)
Other	87 (7.8%)
ART duration in years, median (IQR)	1.0 (IQR: 1.0, 1.0)
0 to 1	856 (77.1%)
2 to 4	137 (12.3%)
≥ 5	118 (10.6%)
*Characteristics at TB diagnosis*	
Calendar year of TB diagnosis	
2008_2010	376 (33.8%)
2011_2012	430 (38.7%)
2013_2014	305 (27.5%)
Hepatitis B status prior/during TB treatment	
Negative	728 (94.3%)
Positive	44 (5.7%)
Anatomic site of TB	
Pulmonary TB	649 (59.2%)
Extra pulmonary TB	448 (40.8%)
Type of TB diagnosis	
Microbiological	400 (36.0%)
Clinical	711 (64.0%)
TB history	
First episode	956 (86.0%)
Retreatment	155 (14.0%)

Missing values: CD4 count (n = 185), Body mass index (n = 61), WHO stage (n = 6), Hepatitis B (n = 339) Anatomic site of TB (n = 14). Of the pulmonary TB cases, N = 649: Pulmonary, smear negative, n = 268, Pulmonary, smear positive, n = 381.

### All-cause mortality post TB treatment

**Fig. 2:**
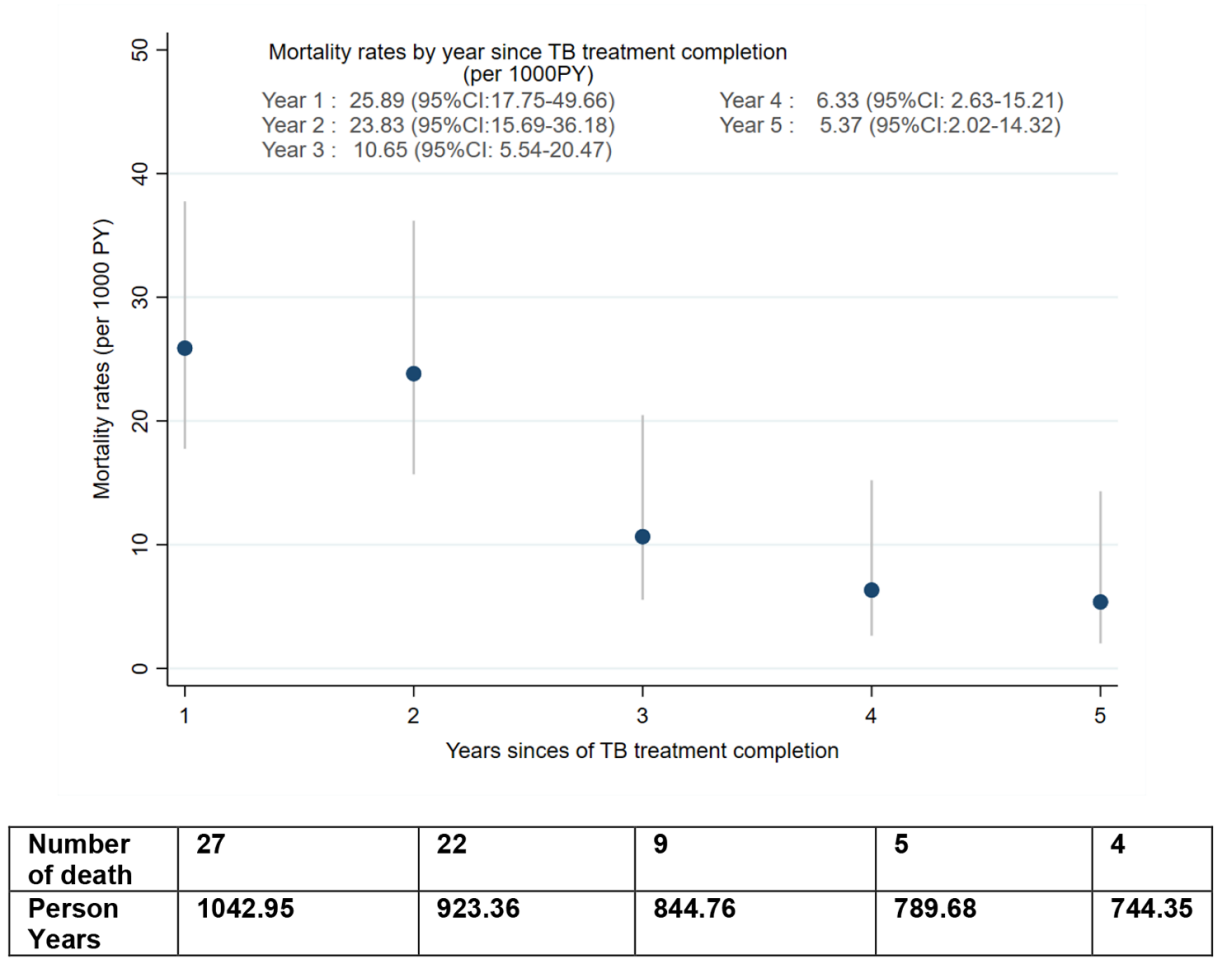
Mortality rates per 1000 Person-Years by year post TB treatment completion

The average follow-up time from TB treatment completion was four years and the total person-time at risk was 4,410.60 person-years (PY). Of the 1111 patients 159 (14.3%) were loss to follow-up, 205 (18.5%) were transferred-out, 67 (6%) died, and 680 (61.2%) were administratively censored. The overall all-cause mortality rate was 15.42 (95%CI: 12.14–19.59) per 1000 person-years. The mortality rate at one, three, and five years was 25.89 (95%CI: 17.75–49.66) per 1000 person-years, 10.65 (95%CI: 5.54–20.47) per 1000 person-years, and 5.37 (95%CI: 2.02–14.32) 1000 person-years, respectively (Fig. 2).

The rate of all-cause mortality varied by patient’s characteristic and was notably higher in patients with a body mass index < 18KgM^2^, < 200cells/mL, 16–24 year-olds, Clinical Stage IV, and those with a history of TB treatment (Table S1). In the Kaplan-Meier analysis, the cumulative probability of death at one year was 2.6% (95%CI: 1.8 − 3.7), 5.8% (95%CI: 4.5 − 7.5) at three years, and 6.9% (95%CI: 5.5- 8.8) at five years (Fig. 3A). By patient’s characteristics, the cumulative probability of death was higher among patients with a CD4 cell counts < 200 cells/mL compared to those with CD4 cell count ≥ 200 cells/mL (Log-rank test, *p* = 0.01). Also, the probability of death was higher among patients with a history of multiple episodes of TB (re-treatment) compared to those with a single episode of TB (first episode), Log-rank test, *p* = 0.02 (Fig. 3B).

**Fig. 3A:**
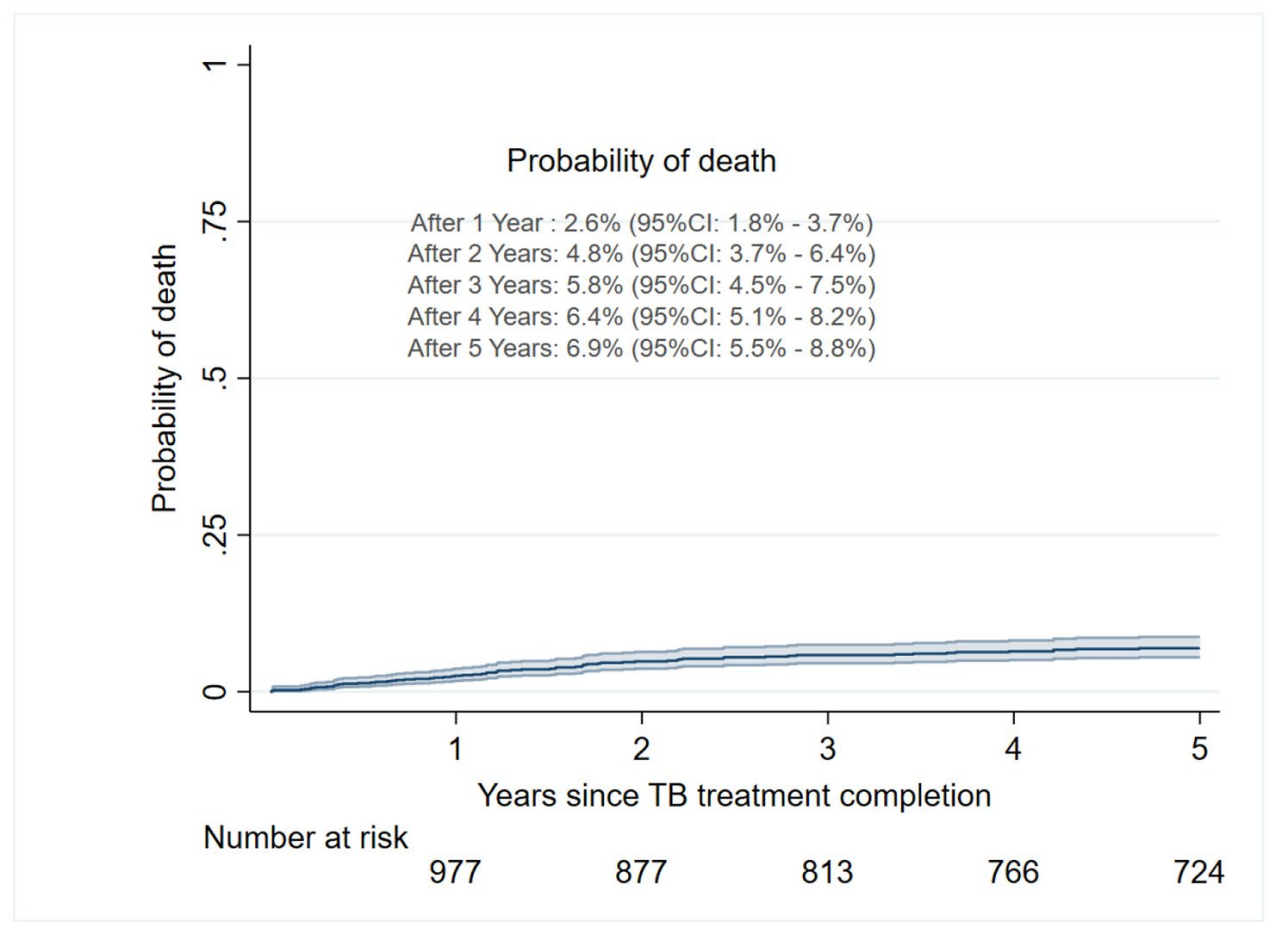
Kaplan-Meier for cumulative probability of death of all patients over time

**Fig. 3B:**
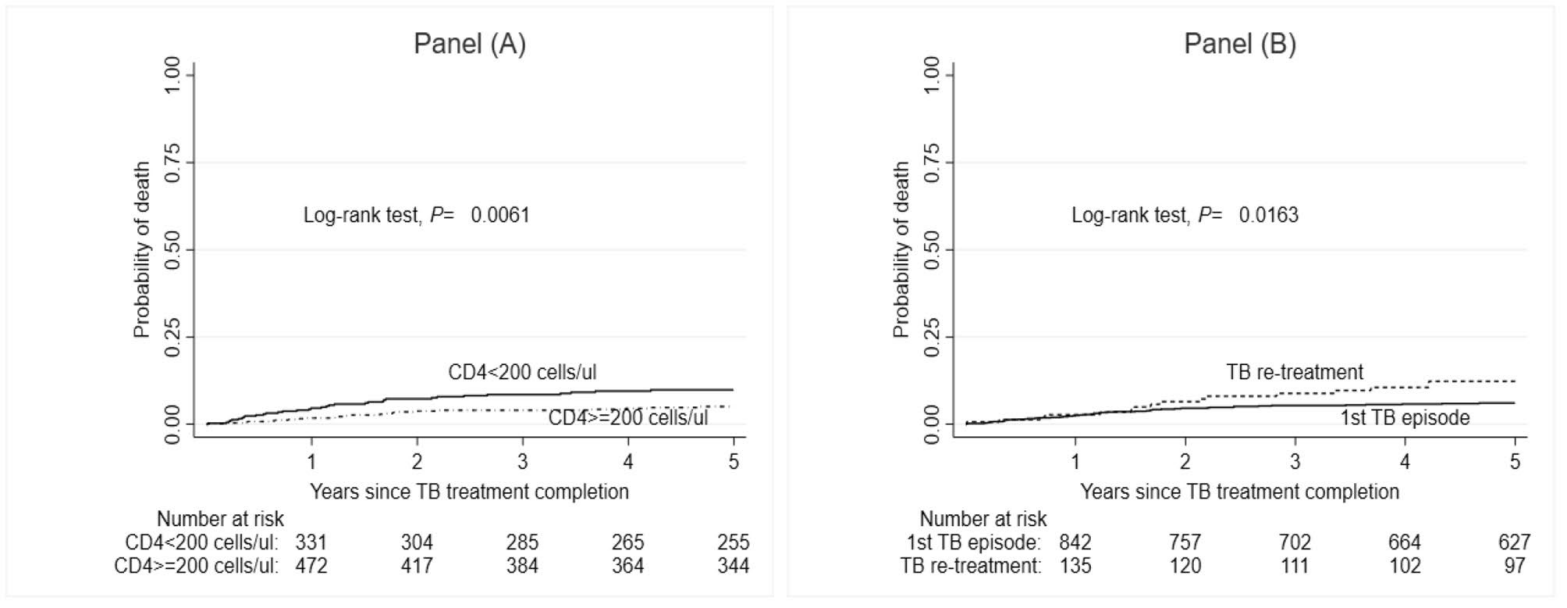
Kaplan-Meier for the cumulative probability of death by CD4 counts (Panel **A**) and TB history (Panel **B**)

In the sensitivity analysis, when all lost to follow-ups were considered as deaths, the mortality rate was 44.65 (95%CI 38.79–51.39) per 1000 person-years which is higher compared to the main analysis where lost to follow-ups were censored (mortality rate 15.42 (95%CI: 12.14–19.59 per 1000 PY). Similarly, the cumulative probability of death at five years was higher 19.2%, (95%CI: 17.0 − 21.7) compared to the main analysis 6.9%(95%CI: 5.5 − 8.8) when all lost to follow-ups were considered dead (Figure S1). In the best case scenario when patients lost to follow-up were considered alive but in care elsewhere, the estimated mortality rate was similar to the main analysis at 14.14 (95%CI 11.13–17.96) per 1000 person-years.

### Factors associated with all-cause mortality post TB treatment

In the multivariable Cox proportional hazard regression model (Table 2), after adjusting for sex, calendar year of TB diagnosis, TB history, WHO HIV clinical stage, and CD4 counts at TB treatment completion, CD4 count less than 200 cells/mL were significantly associated with death (aHR = 1.81, 95%CI:1.06–3.11, *p* = 0.03). Additionally, having TB retreatment history was significantly associated with death (aHR = 2.12, 95%CI: 1.16–3.85, *p* = 0.01). In the multivariable Cox PH regression, 17% (190/1,111) had missing data on at least one of the covariates in the adjusted model. After multiple imputations of missing data on covariates in the adjusted model, CD4 counts <200 cells/mL and TB retreatment history remained significantly associated with an increased risk of death (aHR = 1.78, 95%CI: 1.06–2.97, and 1.87, 95%CI: 1.06–3.30, respectively). Also, after multiple imputations, WHO HIV clinical stage IV at TB treatment completion became significantly associated with death (aHR = 1.85, 95%CI: 1.08–3.17) (Supplementary, Table S2).

**Table 2:** Cox proportional hazard model for mortality after TB treatment completion

Characteristics	Unadjusted HR (95%CI)	*p-*value	Adjusted HR† (95%CI)	*p*-value
*Characteristics at TB treatment completion*				
Sex				
Female	1		1	
Male	1.47 (0.90–2.39)	0.122	1.60 (0.93–2.75)	0.093
Age (in years)				
16–24	1		**–**	
25–34	0.55 (0.23–1.29)	0.170	**–**	
35–44	0.57 (0.24–1.31)	0.183	**–**	
45+	0.77 (0.31–1.89)	0.571	**–**	
WHO HIV stage				
Stage III	1		1	
Stage IV	1.85 (1.09–3.16)	0.023	1.58 (0.90–2.78)	0.113
CD4 counts at TB treatment completion (cells/mL)				
< 200	2.06 (1.22–3.50)	0.007	1.81 (1.06–3.11)	0.030
≥ 200	1		1	
Body mass index (Kg/M^2^)				
< 18	3.79 (2.04–7.05)	< 0.001	**–**	
≥ 18	1		**–**	
ART duration (years)	0.98 (0.87–1.11)	0.752		
ART regimen type				
First-line	1		**–**	
Other	0.93 (0.38–2.32)	0.882	**–**	
*Characteristics at TB diagnosis*				
Year of TB diagnosis				
2008–2012	1		1	
2013–2014	0.57 (0.30–1.06)	0.074	0.61 (0.31–1.23)	0.167
Type of TB diagnosis				
Microbiological	1		**–**	
Clinical	1.70 (0.93–3.12)	0.084	**–**	
TB history				
First episode	1		1	
Retreatment	1.97 (1.12–3.45)	0.018	2.12 (1.16–3.85)	0.014
Anatomic site of TB				
Pulmonary TB	1		**–**	
Extra-pulmonary TB	1.26 (0.78–2.04)	0.340	**–**	

HR denoted Hazard ratio, CI confidence interval.

†Analysis performed on complete cases (n = 921). Factors which had *p-* value < 0.2 were included in the adjusted model and removed if *p-* value > 0.2. Body mass index was removed because it was highly collinear with CD4 counts and TB history.

## Discussion

To the best of our knowledge, this is the first study in sub-Saharan Africa to investigate the survival of ART experienced patients on long term co-trimoxazole prophylaxis that complete TB treatment. In this cohort, mortality is highest in the first two years after TB treatment completion. It then decreased drastically and was lowest in the fifth year post TB treatment. Similarly, the observed probability of death virtually doubled by year two and nearly plateaued after year three. The observation that most deaths post-TB treatment occur within two years of treatment completion could be attributed to; sequelae of severe TB disease, residual lung damage, [7] recurrent bacterial infections post TB, [19] and complications of poor immune recovery caused by HIV/TB coinfection. [20, 21].

Compared to other TB burden countries, the observed long term mortality rate (15.42 per 1000 person-years) was generally lower than has been previously reported in Ethiopia, and China. [22, 23] Similarly, the adjusted 5-year mortality is lower than that in HIV/ TB co-infected cohorts in Latin America, and Myanmar. [15, 24] The better survival in our study may be attributed to IDI being a relatively well supported specialist referral clinic, early adoption of the WHO one-shop model of integrated services delivery, [15] and recent advances in treatment and monitoring of PLHIV. However, in the sensitivity analysis the probability of death almost tripled at five years (19.2% 95%CI: 17.0-21.8) when all lost to follow-ups were considered dead, an indication that survival may be worse after TB treatment despite being on ART.

After adjusting for confounding in the multivariable Cox proportional hazard model, CD4 count < 200 cells/mL was significantly associated with an 81% increased risk of death. This finding is consistent with the literature. [14] The association with mortality could be that patients with a low CD4 count after TB treatment fail to achieve robust immune recovery despite being on ART, and thus remain susceptible to several opportunistic infections even with virological suppression. [21] Furthermore, these patients may be more susceptible to complications from chronic inflammation due to TB and HIV replication. Such chronic inflammation leads to cardiovascular compilations which have been identified as a cause of death after TB treatment. [10, 11] Additionally, having a history of TB retreatment was significantly associated with a 200% increased risk of death after TB treatment compared to those with a single episode of TB. This could probably be due the cumulative residual damage left by each TB episode in these patients, [25] and hence a reduction in the survival. [10] Additionally, TB recurrence in those with a history of TB could indicate ongoing immune dysfunction, [26, 27] and hence an increased risk of mortality from AIDS and non-AIDS events in this sub-group. [28]

The findings in this study are generalisable to HIV and TB programs worldwide and highlight the long term prognosis after completion of TB treatment in ART experienced patients. HIV programs and clinics should closely monitor patients in the first two years after treatment completion. During this two-year period, attention should be given to screening for AIDS events, cardiovascular diseases, and pulmonary complications. [28, 29] However, the implementation of such interventions means prioritising certain sub-groups. Such differentiated care should target those with malnutrition, immunological failure, and history of multiple TB episodes. These subgroups may require nutritional counselling and supplementation, use of enhanced prophylaxis, [30] and further screening for opportunistic infections as part of the minimum package immediately after TB treatment. Additionally, the increased probability of death shortly after TB treatment coupled with the known risk of TB recurrence associated with low CD4 [26] underscore the need for clinics and programs to strengthen the coverage and uptake of intermittent preventive therapy in TB survivors. Taken together, the results justify the need to redefined TB treatment success in PLHIV to include a time period and intervention package post TB treatment completion.

As it usually happens with retrospective studies, our study presents some limitations. There is a limited number of variables that could be explored with this clinical dataset. For instance, there was no data on socio-economic variables like education level, occupation status, alcohol use and homelessness which have been associated with long term mortality in some studies. [12, 14] Also, in resource-limited settings some laboratory tests are not regularly performed, for instance, haemoglobin and drug resistance testing yet anaemia and drug resistance may be associated with long term mortality. Similarly, most TB cases were diagnosed clinically; however, TB tends to be atypical and extra pulmonary in HIV patients hence making a laboratory confirmation difficult. [ 27] The study did not have end of treatment chest-radiographs and information on the causes of death. Therefore, it is impossible to quantify the contribution of post-TB lung injury and post TB lung disease to mortality. The study did not collect data on AIDS defining conditions after TB treatment. Moreover, the study was conducted at a single site and this may limit the quality and generalisability of results. Lastly, the study had a lot of transfer outs and lost to follow-ups and the exact outcome of these patients is unknown. Despite the limitations, this observational fixed cohort was sufficiently large with adequate observation and follow-up time of five years and included only patients on lifelong triple ART and co-trimoxazole prophylaxis making the results generalisable to similar settings.

## Conclusion

In ART experienced patients who complete TB treatment, long-term survival is reasonably good. Mortality is highest within the two years post TB treatment completion. Patients with low a CD4 cell count and those with a history of TB retreatment have an increased risk of all-cause mortality and this underscores the need for detailed assessment, close monitoring, and use of enhanced prophylaxis after TB treatment to improve survival. Further research is needed to understand the causes of death, and the contribution of post TB disease to morbidity and mortality.

## Electronic supplementary material

Below is the link to the electronic supplementary material.

Supplementary Material 1

## Data Availability

The datasets used in this study are available from the corresponding author upon reasonable request. However, some access criteria will be applied by the institution’s scientific research committee.

## Abbreviations

aHR: Adjusted Hazard ratios
AIDS: Acquired Immune Deficiency Syndrome
ART: Antiretroviral Therapy
BMI: Body mass index
Cox: PH: Cox proportional hazard
DSPTB: Drug-Sensitive Pulmonary TB
ETB: Extra Pulmonary Tuberculosis
IQR: Interquartile ranges
HIV: Human Immunodeficiency Virus
PLHIV: People Living with HIV
PMTCT: Prevention of Mother to Child Transmission
PY: Person-years
TB: Tuberculosis
uHR: Unadjusted Hazard ratios
WHO: World Health Organization
